# Starling curves and central venous pressure

**DOI:** 10.1186/s13054-015-0776-1

**Published:** 2015-02-16

**Authors:** David A Berlin, Jan Bakker

**Affiliations:** Division of Pulmonary and Critical Care Medicine, Department of Medicine, Weill Cornell Medical College, 1300 York Avenue, New York, NY 10021 USA; Erasmus MC University Medical Center Rotterdam, PO Box 2040 – Room H 625, Rotterdam, 3000 CA the Netherlands

## Abstract

Recent studies challenge the utility of central venous pressure monitoring as a surrogate for cardiac preload. Starting with Starling’s original studies on the regulation of cardiac output, this review traces the history of the experiments that elucidated the role of central venous pressure in circulatory physiology. Central venous pressure is an important physiologic parameter, but it is not an independent variable that determines cardiac output.

## Introduction

This year marks the 100th anniversary of the publication of the Starling curve. The curve demonstrated the relationship between right atrial pressure and cardiac output [1]. Consequently, some clinicians have used central venous pressure (CVP), which is right atrial pressure, to determine the adequacy of circulating blood volume and cardiac preload.

Depictions of Starling curves with CVP on the *x* axis (abscissa) have invited clinicians to presume that fluid resuscitating patients with low CVP would increase the stroke volume and cardiac output. Indeed, important clinical practice guidelines recommend fluid resuscitation of hypoperfused patients until the CVP rises to abnormal levels [2]. A steady accumulation of investigations, however, has challenged this common practice [3]. Initially, a report demonstrated that CVP does not correlate with gold standard measurements of total blood volume [4]. Subsequently, two systematic reviews revealed that CVP does not predict the response of cardiac output to a fluid bolus in critically ill patients [5,6]. Similarly, pulmonary capillary wedge pressure also does not predict the response to fluid administration [7]. These challenges may lead some to take a nihilistic attitude toward hemodynamic monitoring and others to question the validity of Starling’s law of the heart. In fact, the results of these studies are an expected consequence of circulatory physiology [5].

This review traces the history of Starling curves to explore their proper role in circulatory physiology. To use CVP and Starling curves correctly, we have to recognize that Starling’s original experimental model set CVP as the dependent variable of venous return and cardiac function. The model also excluded the effects of an important factor in critically patients – the pressure adjacent to the heart.

## The starling curve

**Figure 1:**
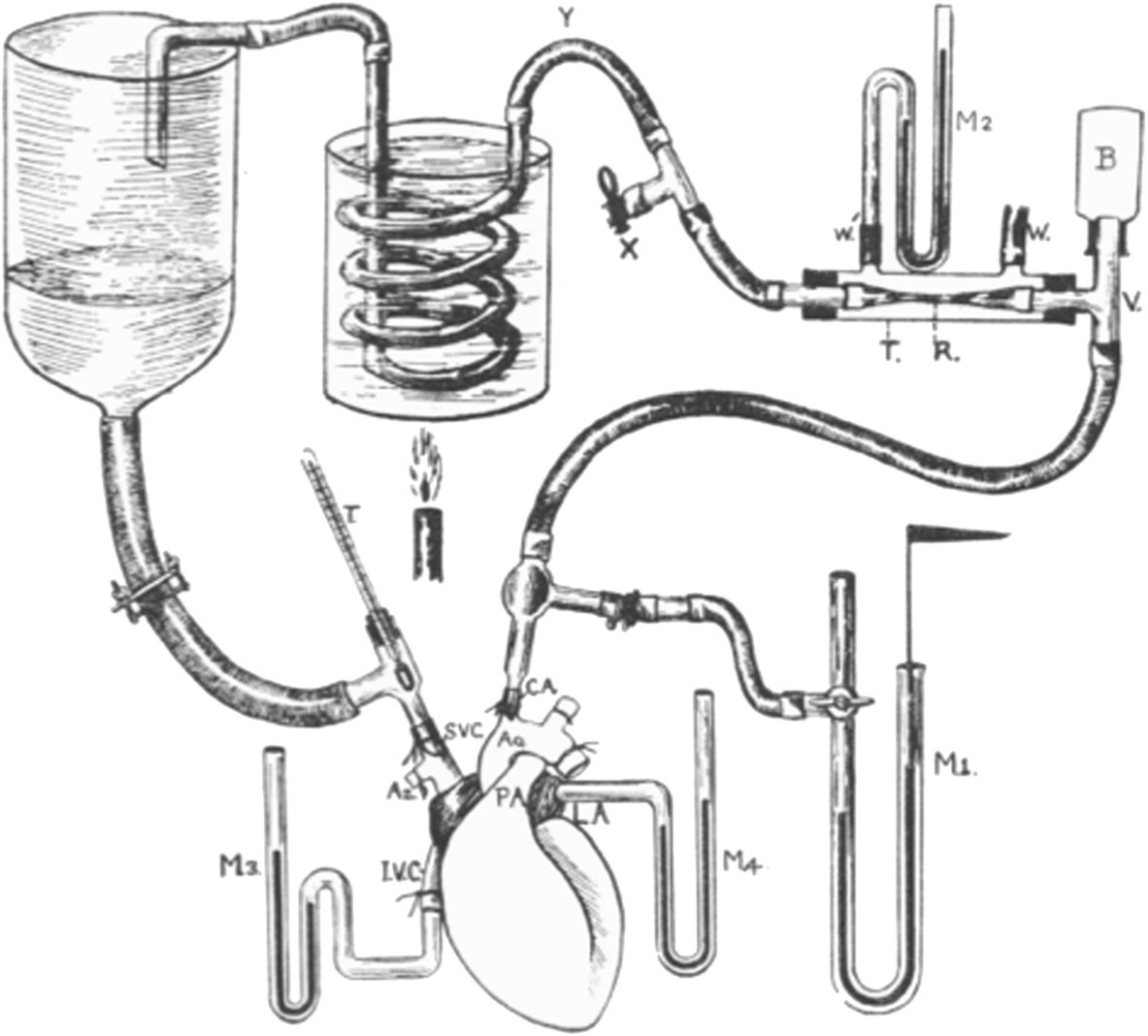
**Starling’s heart–lung preparation.** The coronary and pulmonary circulations are left intact and the lungs are not shown. Image reproduced with permission from [1].

In 1914 Starling used his isolated heart–lung preparation to study cardiac output (Figure 1). He performed thoracotomies on anesthetized dogs during positive pressure ventilation. Leaving the pulmonary and coronary circulations intact, he ligated the inferior vena cava, the distal aorta, and the branches off the aortic arch. A cannula in the aortic arch diverted the systemic flow into an extracorporeal circuit. The left ventricle pumped blood through the circuit into an elevated blood reservoir. Gravity pulled blood out of the reservoir through a cannula leading into the superior vena cava. By slowly opening a clamp placed on the cannula, Starling increased the rate of blood flow from the reservoir into the right atrium. He showed that, over a wide range, the heart ejected whatever volume of blood the system returned to the right atrium. Since the rate of flow into the right atria matched the flow out from the aorta, Starling named both flows cardiac output [1].

**Figure 2:**
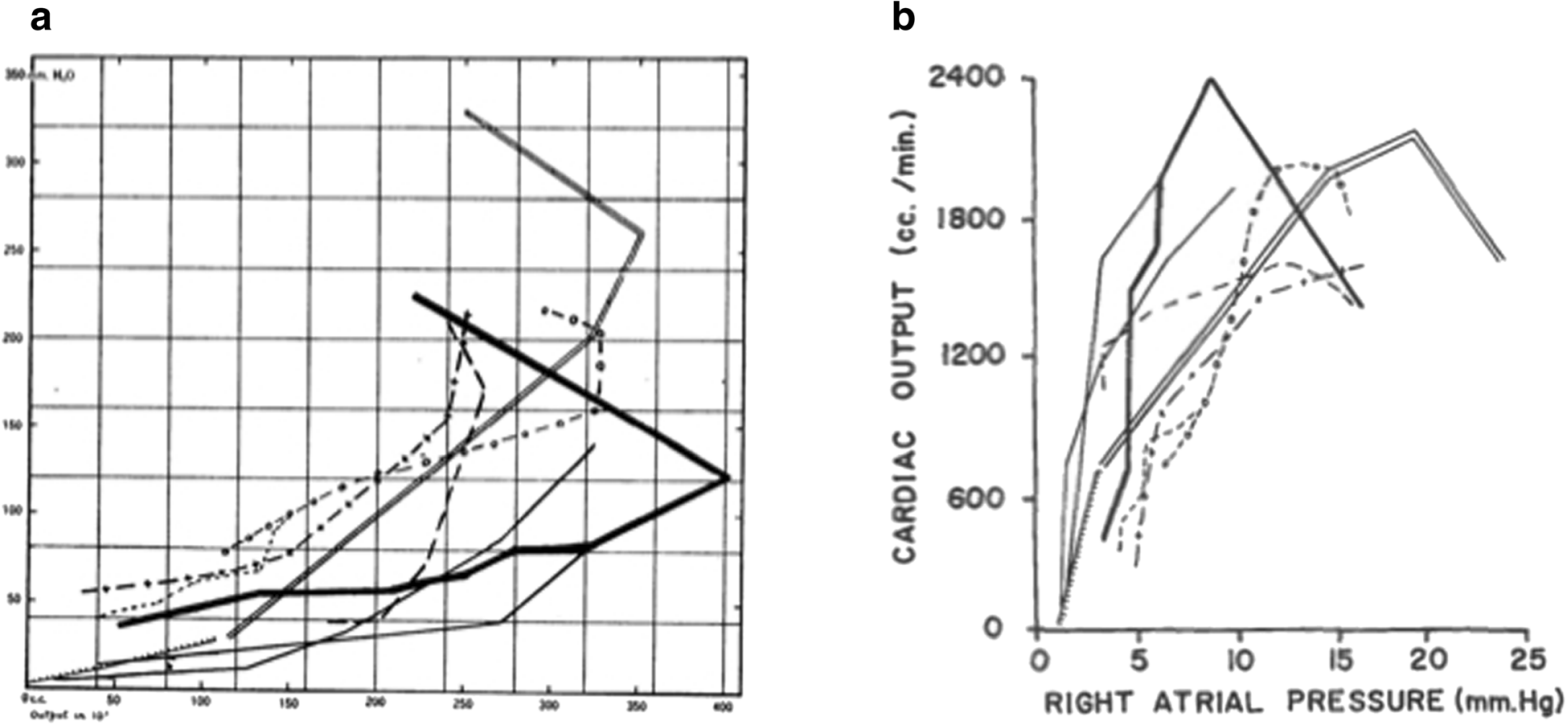
**Original presentation and reproduction of the Starling curve. (a)** Original presentation of the Starling curve. The *y* axis is right atrial pressure (mmH_2_O). The *x* axis is cardiac output (cm^3^/minute). Image reproduced with permission from [1]. **(b)** Reproduction in Guyton and colleagues’ textbook on circulatory physiology. Image reproduced with permission from [9].

As Starling opened the resistor, blood flowed at an increasing rate from the venous reservoir into the right atrium. Initially, right atrial pressure rose slowly. There was a limit to the heart’s capacity to accommodate the increased rate of blood return. Beyond this limit, the heart (in Starling’s words) fatigued. Blood began to well up in the right atrium – rapidly raising right atrial pressure. Starling presented the data from nine experiments in separate curves. There was no test of statistical significance (Figure 2a). Surprisingly, Starling graphed the right atrial pressure on the *y* axis (ordinate) and cardiac output on the *x* axis (abscissa) [1]. Some have claimed that Starling and his editor were unaware of the custom of placing the independent variable on the *x* axis [8]. In subsequent reproductions, other authors flipped the curve to place right atrial pressure on the *x* axis (Figure 2b) [9]. Starling’s text, however, does not suggest that right atrial pressure is an independent variable that controls stroke volume or myocardial work. Starling’s experimental variable was the amount he opened the resistor on the cannula that carried blood back to the heart. Starling emphasized that right atrial pressure rose as a consequence of increased blood return to the heart. He interpreted this finding to mean that the heart could accommodate varying amounts of blood return, up to a physiologic limit. As he increased the blood return beyond the limit of accommodation, blood dammed up in the heart and cardiac output fell. A rapid rise in right atrial pressure signaled that circulation had exceeded the heart’s limit of accommodation [1].

**Figure 3:**
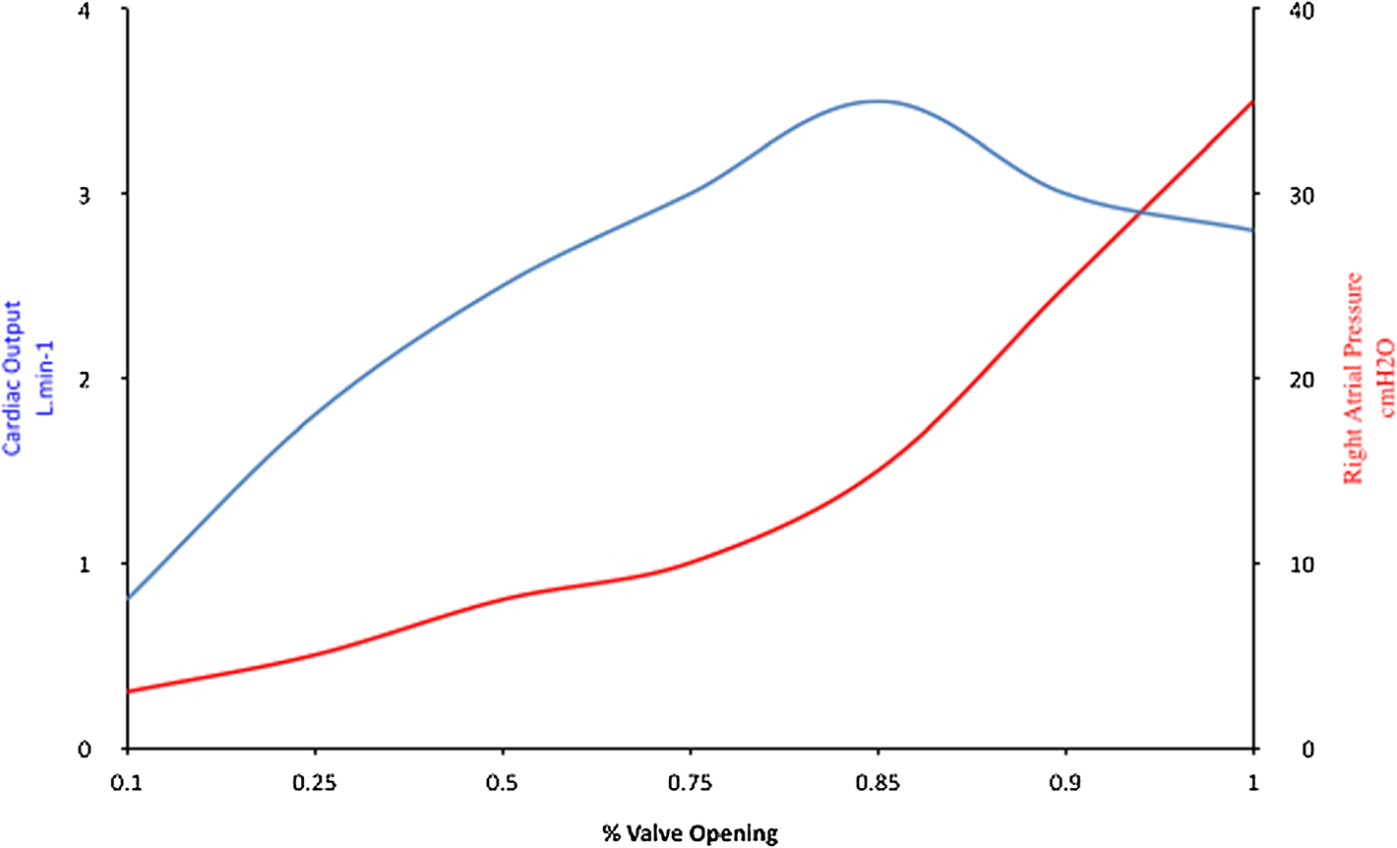
**Alternative presentation of the Starling curve.** Alternative presentation of the Starling curve with the actual independent variable (opening of the venous return valve) on the *x* axis. The original raw data from the experiments is unavailable.

Starling’s graph of cardiac output and right atrial pressure is somewhat confusing to the modern reader. Neither one of these two variables was independent in his experiment. The actual independent variable of his experiments was the amount he opened the valve that resisted flow into the right atrium. Using the modern custom, the absence of resistance to blood return (the amount of valve opening) would appear on the x axis. The dependent variables cardiac output and right atrial pressure would appear on separate *y* axes (Figure 3). This graph demonstrates the intrinsic ability of mammalian hearts to respond to changes in the rate of returning blood flow. Once the rate of blood return exceeds the limit of the heart’s capacity to respond, right atrial pressure rises rapidly. Beyond the limit of accommodation, cardiac output plateaus or falls. The Starling curve does not imply that increasing right atrial pressure increases cardiac output. In some experiments, Starling increased resistance to outflow from the aorta. The increased load raised right atrial pressure without increasing cardiac output [1].

Apparently, Starling never shared the enthusiasm for his curve. He did not mention it in his famous Linacre lecture on the heart, which integrated his extensive experiments into a cohesive framework [10]. Nor did he show the curve in his textbook of medical physiology. For Starling, the curve was just part of the basis for the more important insight: The Law of the Heart. Simply stated, myocardial work varies with the initial fiber length of the myocyte [10,11]. The dominant role of thermodynamics in the study of physiology in that era explains Starling’s emphasis on myocardial work and heat generation.

## Other starling curves

**Figure 4:**
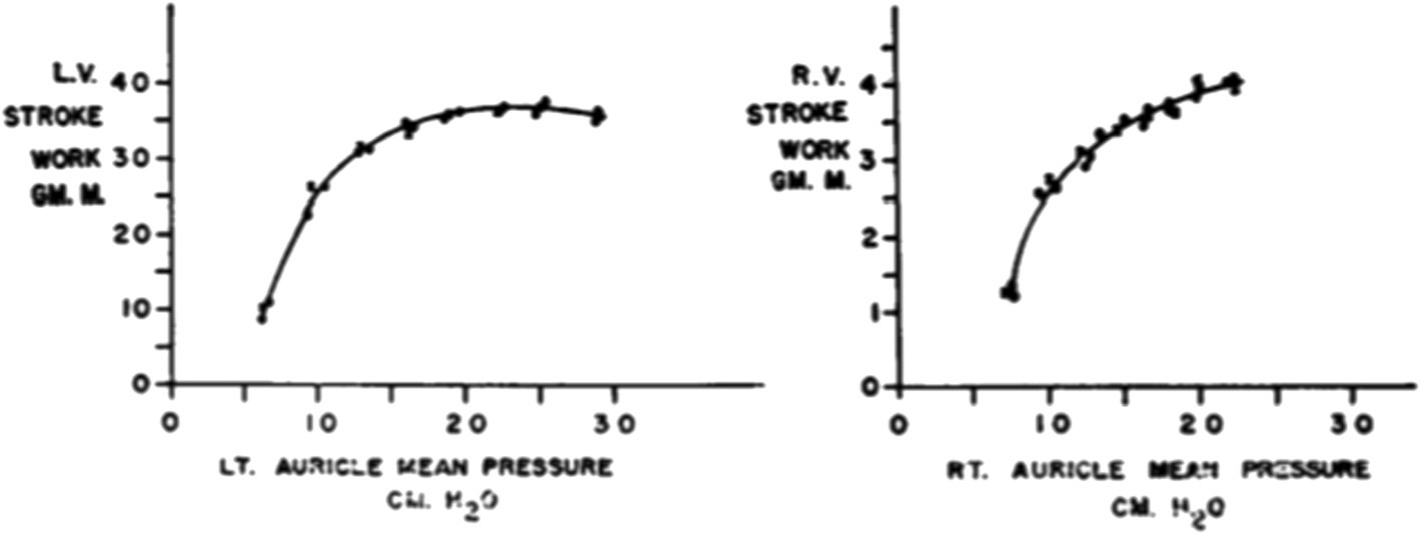
**Sarnoff’s curves.** The atrial pressures reflect filling in these experiments. L.V., left ventricular; R.V., right ventricular. Image reproduced with permission from [12].

Forty years after Starling published his curve, Sarnoff and Berglund demonstrated the Law of the Heart with a more intact systemic circulation. They performed thoracotomies on anesthetized dogs on positive pressure ventilation and cannulated the aorta and both atria. They filled the right atrium with blood and fluids from elevated blood reservoirs. They measured ventricular work and cardiac output by an electromagnetic flow meter on the aorta. As they increased blood return to the heart, the myocardial work and stroke volume increased up to a physiologic limit. They graphed their findings with atrial pressure on the *x* axis, a pattern that is more familiar to modern clinicians (Figure 4) [12]. Sarnoff and Berglund manipulated atrial pressure with elevated reservoirs in an open chest model. In these experiments, atrial pressure was a determinant of preload since the height of the reservoir increased the driving pressure of blood return to the ventricles. In other experiments, they demonstrated the absence of a direct relationship between absolute right atrial pressure and stroke work in the presence of elevated intrathoracic or pleural pressure. In these situations, elevated right atrial pressure is not due to distention with blood. Instead, as right atrial pressure rises, stroke volume falls [13]. These experiments reveal that the measured CVP is a composite of the pressure generated by the volume of blood that tends to distend the right atrium and the pressure in the pericardium and thorax adjacent to the heart. The pressure adjacent to the heart (called juxta-cardiac pressure) is negligible in experimental conditions of an open chest and pericardium. Much of the confusion in clinical settings about CVP arises from a failure to consider sources of significant juxta-cardiac pressures.

Sarnoff and Berglund’s work revealed another limitation to using CVP as a determinant of stroke volume. They decreased myocardial compliance by increasing ventricular afterload and inducing coronary ischemia. These interventions increased right atrial pressure without increasing the stroke volume. The investigators summarized their extensive experiments by stating that for each animal there is a family of curves that describe the relationship between atrial pressure and cardiac work and output [14]. They never intended to imply that right atrial pressure is an independent variable which controls cardiac output.

**Figure 5:**
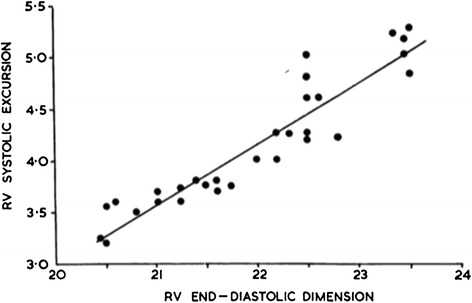
**Ventricular excursion varying directly with filling by negative pressure inspiration.** RV, right ventricular. Image reproduced with permission from [15].

In the 1960s Braunwald and colleagues evaluated the Starling law in human patients. This group sewed metallic markers into ventricles and measured ventricular volume by cineradiography. Braunwald used the large beat-to-beat variation in filling during atrial fibrillation to show that stroke volume varies as a function of ventricular end-diastolic volume. Similarly, he showed that right ventricular systolic excursion varied with ventricular filling during valsalva maneuvers (Figure 5). This curve demonstrates that filling and stretch of the ventricle increases the stroke volume. This experiment shows how stroke volume varies with right atrial pressure. Deep inspiration lowers intra-thoracic pressure, thereby lowering right atrial pressure. Reduced right atrial pressure increases ventricular filling and augments stroke volume [15].

All of the preceding experiments confirmed that the heart has the intrinsic ability to accommodate changes in preload; increasing diastolic volume and stretch increase the force of contraction. Elevated cardiac pressure only increases the force of contraction and stroke volume if the experimental conditions permit the filling pressure to be an accurate proxy for end-diastolic volume. Cardiac tamponade, positive pressure ventilation, and increased ventricular afterload all increase atrial pressure without increasing the stroke volume [1,13,15,16]. Much of the confusion about the relationship between CVP and Starling curves arose from its graphic depiction. Since CVP is often depicted on the *x*-axis, some clinicians incorrectly assume that it is an independent variable. However, CVP is only a surrogate for the return of blood to the heart under a set of defined circumstances that are not consistently present in clinical situations.

## Guyton’s curves

A more complete understanding of the relationship between CVP and Starling’s law required knowledge of the forces that return blood to the heart. During the 1950s to 1970s, Guyton integrated these features into a comprehensive model of the circulation and the control of cardiac output. Guyton confirmed Starling’s belief that the metabolic activity of the peripheral organs is a key factor regulating the rate of venous return to the heart. According to Guyton’s model, venous return depends on the pressure gradient between the average vascular pressure (called the mean systemic filling pressure) and the right atrium. Therefore, an elevated right atrial pressure opposes venous return. CVP is the clinical measurement of right atrial pressure. The gradient between the mean systemic filling pressure and CVP creates venous return and cardiac output. The mean systemic filling pressure arises from the force acting between the blood and the walls of the blood vessels and is largely independent of pulsatile left ventricular contraction [17].

**Figure 6:**
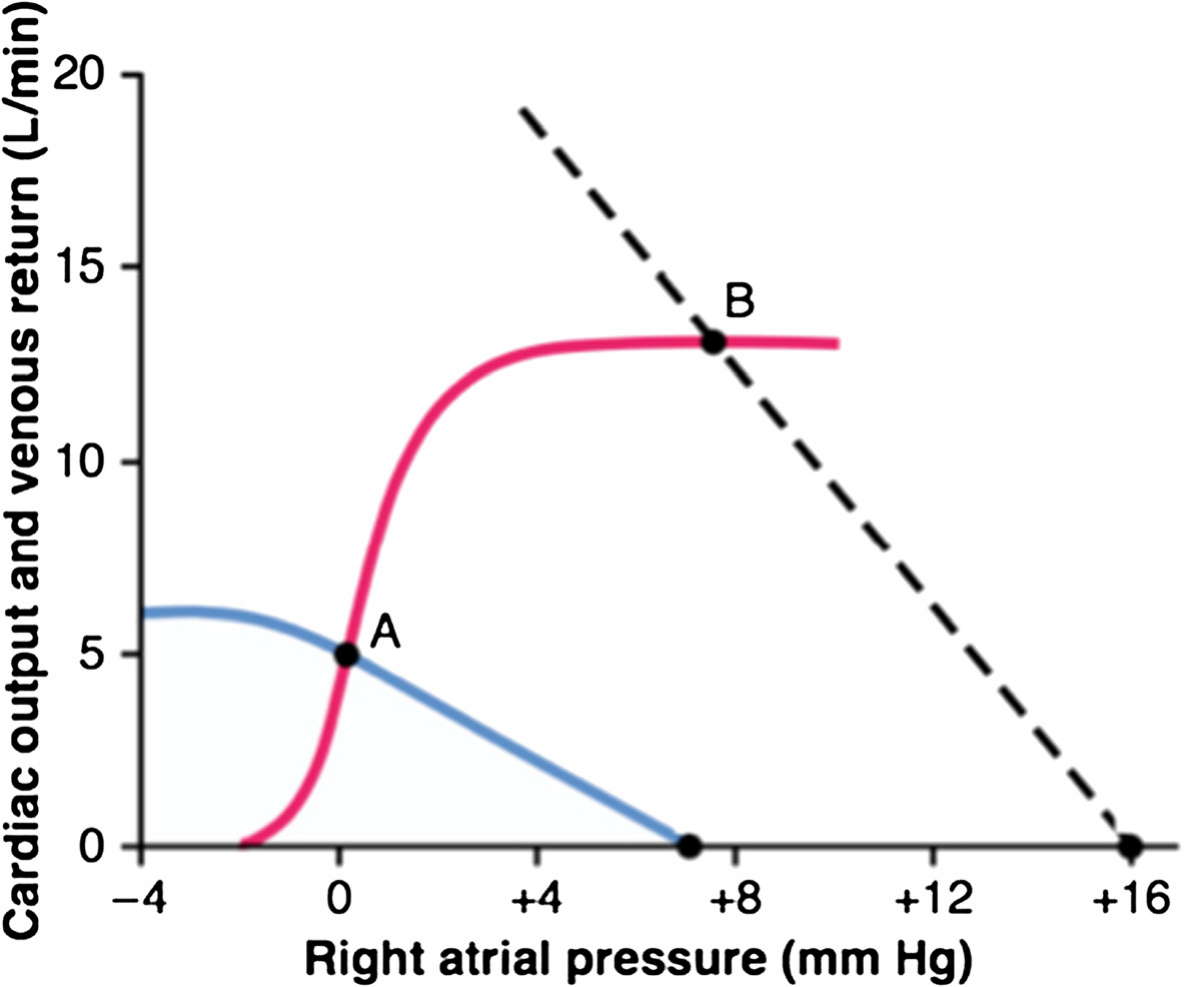
**Guyton’s combination of two curves to model the circulation.** The intersection of the red cardiac performance and blue venous return curves determines cardiac output. Point A is the normal rest situation. Cardiac output is higher when the dashed line is the venous return curve and intercepts the same cardiac performance curve at point B. Image reproduced with permission from [30].

Guyton used the combination of two curves to model the circulation. The intersection of these two curves determines the cardiac output (Figure 6). The venous return curve shows the rate of blood return to the heart and depends on the mean systemic filling pressure, right atrial pressure, and vascular resistance. An increase in vascular pressure that drives blood into the heart shifts the curve upward. The sinusoidal cardiac function curve depends on ventricular afterload, autonomic tone, and many intrinsic factors that determine cardiac performance. The cardiac function curve would be rotated downward and to the right in a poorly performing heart and intersects the venous return curve at a lower rate of cardiac output. The cardiac performance curve is analogous to a Starling curve. For a given cardiac performance, increasing venous return intersects the cardiac performance curve at a higher point and results in higher cardiac output. This is a graphic representation of Starling’s Law of the Heart. The plateau of the cardiac performance curve shows that there is a limit to the heart’s ability to accommodate increases in venous return. If venous return increases beyond this limit, blood wells up in the heart and raises atrial pressure drastically. Moreover, if a given venous return curve intercepts a cardiac performance curve from an ineffective heart, cardiac output is lower and right atrial pressure is higher. This is precisely what Starling showed: if increasing amounts of blood are returned to a fatigued heart, atrial pressures rise as cardiac output falls.

Guyton’s curves allow us to do what Starling initially intended for his curve; to help predict the response of the entire circulation to changing a single variable. The curves of Starling and Sarnoff and Berglund are the results of experiments designed to study the response of the heart. Guyton’s model incorporates the findings of these investigators into a holistic framework. Of course, the complexities and compensatory responses of the circulatory system prevent simple prediction of the response in cardiac output. In patients, the individual determinants of cardiac output are deeply interrelated. We simply cannot change just one variable (such as CVP) and predict a response in cardiac output. As we have seen, CVP depends on cardiac output as much as it determines it. Even though Guyton placed right atrial pressure (CVP) on the *x* axis, it is not an independent variable that determines cardiac output.

## Clinical use of central venous pressure and Starling’s law

As we have seen, the Starling curves that describe the Law of the Heart do not justify intentionally raising CVP with fluid resuscitation to abnormal values to increase cardiac output. However, the fact that an isolated measurement of CVP does not predict the response to a fluid bolus does not mitigate its importance as a hemodynamic variable. An understanding of Starling curves provides a rationale for using CVP as part of the evaluation of hemodynamic instability. First, an elevated CVP may signify that there is an impediment to venous return. The clinician should search for elevated juxta-cardiac pressure. These causes include tension pneumothorax, cardiac tamponade, and excessive positive pressure ventilation. Importantly, high CVP can be a sign of unrecognized auto-positive end-expiratory pressure due to dynamic hyperinflation, which may lead to shock and death. When high juxta-cardiac pressures impede venous return, clinicians should consider fluids and venoconstrictors to restore the gradient for venous return [18].

As the Starling curve shows, elevated CVP can also be an indication that venous return has exceeded the limit of cardiac accommodation. While CVP may be normal in left heart failure and pulmonary edema [19], an elevated CVP may signify that blood is welling up in the right atrium due to right heart dysfunction or obstruction to right ventricular outflow. CVP elevation, not reduced cardiac index, is the hemodynamic variable that correlates best with renal failure and hepatic dysfunction in congestive heart failure. An elevation of CVP to 4 mmHg is associated with an increased risk of death [20]. Elevated CVP is probably a marker for hepatic and renal congestion in heart failure [20-23]. An elevated CVP is also the hemodynamic parameter best associated with the development of renal failure in severe sepsis [24,25].

CVP elevation (in the absence of elevated juxta-cardiac pressure or tricuspid valve disease) is an important marker of right heart failure due to elevated pulmonary vascular resistance. This syndrome is common among critically ill patients and is often due to acute pulmonary emboli or the acute respiratory distress syndrome [26,27]. A rise in CVP in these situations is arguably a more important finding than pulmonary artery hypertension. As pulmonary vascular resistance rises beyond the limits of right ventricular compensation, the pulmonary artery pressure will fall as cardiac output falls. CVP, however, will continue to rise into double digits as blood wells up in the right side of the heart [28]. The rise in CVP coincides with a rapid increase in right ventricular systolic and diastolic pressure, which can ultimately culminate in a diminution of the coronary perfusion gradient to the right ventricular myocardium [27].

## Conclusion

The preceding paragraphs enumerate examples of diagnostic considerations when CVP elevation occurs in the setting of hypoperfusion. Similarly, normal CVP evidences against the presence of right ventricular pressure overload or elevated juxta-cardiac pressure. Clinicians should also consider using CVP measurement because it can be obtained non-invasively. Ultrasound measurement of the inferior vena cava reliably predicts CVP during spontaneous (negative pressure) ventilation [29]. To understand CVP, we must recognize its relation to venous return and the Law of the Heart. An isolated measurement of CVP, like any other single hemodynamic variable, cannot describe the state of the circulation. However, when history and physical examination are insufficient, clinicians can integrate CVP into hemodynamic assessment along with other monitoring such as functional hemodynamics and echocardiography.

The small sample size and absence of statistical analysis in Starling’s 1914 article would probably not pass peer review today. However, the subsequent century of research has validated the curve. Despite enduring confusion over its axes and its relationship to CVP, the Starling curve deserves its prominent place in our understanding of circulatory physiology.

## Abbreviation

CVP: Central venous pressure
